# Prognostic significance of liver stiffness in patients with primary biliary cholangitis: validation of Baveno VII criteria

**DOI:** 10.1007/s12072-023-10587-w

**Published:** 2023-09-19

**Authors:** Dawei Ding, Guanya Guo, Lina Cui, Gui Jia, Xiufang Wang, Miao Zhang, Siyuan Tian, Linhua Zheng, Yansheng Liu, Yinan Hu, Guoyun Xuan, Jiaqi Yang, Chunmei Yang, Ruiqing Sun, Juan Deng, Changcun Guo, Yu Chen, Yulong Shang, Ying Han

**Affiliations:** 1 State Key Laboratory of Cancer Biology, Xijing Hospital of Digestive Diseases, The Air Force Military Medical University, Xi’an, 710032 Shaanxi China; 2 Fourth Department of Liver Disease (Difficult & Complicated Liver Diseases and Artificial Liver Center), Beijing You’an Hospital Affiliated to Capital Medical University, Beijing, China

**Keywords:** Primary biliary cholangitis, Liver stiffness measurements, Prognosis, Retrospective cohort study

## Abstract

**Background:**

The role of liver stiffness measurements (LSM) in patients with primary biliary cholangitis (PBC) remains to be further elucidated.

**Aims:**

To clarify the prognostic role of LSM and to validate the “novel concepts” proposed by the Baveno VII Working Group.

**Methods:**

An analysis of the prognostic significance of LSM was performed involving 672 patients.

**Results:**

LSM and ΔLSM/ΔT were independent risk factors for liver decompensation, liver transplantation, or liver-related death (primary outcomes, *p* < 0.001, both). A rule of 5 kPa for LSM (10–15–20 kPa) could be used to denote progressively higher relative risks of primary outcomes. Patients with LSM < 10 kPa have a negligible 3-year risk of primary outcomes (< 1%). Cut-off values of 10 and 15 kPa can be used to classify PBC patients into low-, medium-, and high-risk groups. A clinically significant decrease in LSM, evaluated at 6, 12, or 24 months elastography tests, was associated with a substantially reduced risk of primary outcomes (*p* < 0.05, all), which can be defined as a decrease in LSM of >  − 20% associated with LSM < 20 kPa or any decrease to LSM < 10 kPa. A clinically significant increase in LSM, evaluated at 6, 12, or 24 months elastography tests, was associated with a substantially raised risk of primary outcomes (*p* < 0.05, all), which can be defined as an increase in LSM of ≥  + 20% or any increase to LSM ≥ 15 kPa.

**Conclusions:**

LSM can be used to monitor disease progression and predict long-term prognosis in patients with PBC.

**Supplementary Information:**

The online version contains supplementary material available at 10.1007/s12072-023-10587-w.

## Introduction

Primary biliary cholangitis (PBC) is a slowly progressive disease with a natural course of 10–15 years, but high-risk patients can progress rapidly to decompensated cirrhosis and even death [1]. Early identification of such patients and timely initiation of drug therapy may improve long-term prognosis.

According to different response criteria and prognostic scoring models, biochemical response to ursodeoxycholic acid (UDCA) is usually assessed at 6 months (Ehime [2]), 12 months (Barcelona [3], Paris I [4], Paris II [5], Rotterdam [6], United Kingdom [UK]-PBC [7] and GLOBE [8]) and 24 months (Toronto [9]) after UDCA treatment. Our group retrospectively analyzed 569 PBC patients and developed an early (after 1 month of UDCA treatment) assessment of biochemical response with alkaline phosphatase (ALP) ≤ 2.5 × upper limit of normal (ULN), aspartate aminotransferase (AST) ≤ 2 × ULN, and total bilirubin (TB) ≤ 1 × ULN [10]. The criteria can be used to screen for high-risk cases, making it more likely that patients will benefit from the prompt addition of second-line therapy.

Liver stiffness measurements (LSM) by transient elastography technique, have been shown in several studies to be of prognostic value in patients with PBC. Corpechot et al. [11] found that PBC patients had a fivefold increased risk of liver failure, liver transplantation or death when LSM were > 9.6 kPa. In addition, worsening liver stiffness was associated with an increased risk of adverse outcomes. When the cut-off value of 2.1 kPa/year was taken, patients had an 8.4-fold higher risk of liver failure, liver transplantation or death [11]. An international multicenter study of 3985 PBC patients from 12 countries found that baseline LSM at cut-off values of 8 and 15 kPa allowed for the classification of PBC patients into low-, medium-, and high-risk groups, facilitating the early implementation of individualized interventions for patients in different risk strata [12].

In 2021, the Baveno VII Working Group [13] first proposed the “Rule of 5 kPa”, defined as 5 kPa for LSM (10–15–20–25 kPa) should be used to represent the relative risk of progressively increasing decompensated events and liver-related deaths regardless of the etiology of chronic liver disease (B.1); the first proposal that patients with chronic liver disease with LSM < 10 kPa have a very low risk of decompensation or liver-related death within 3 years (≤ 1%, A.1); and the first proposal for a clinically significant decrease in LSM (CSDL), defined as a reduction in liver stiffness of at least 20% and < 20 kPa, or to less than 10 kPa, was associated with a significant reduction in the risk of patient decompensation or liver-related death (C.2). These “new concepts” have yet to be validated in patients with PBC. Therefore, this study was designed to assess the predictive value of LSM for long-term prognosis in patients with PBC through a retrospective cohort analysis and to validate the “novel concepts” proposed by the Baveno VII Working Group.

## Methods

### Study population

Patients attending the Xijing Hospital of the Air Force Military Medical University from January 2016 to January 2022 who met the inclusion and exclusion criteria were retrospectively included in the study. In total, 672 patients with PBC and 2552 valid LSM were included. The average number of LSM per patient was 3.2 ± 2.1 (interquartile range [IQR], 1–5). The average time interval between two successive LSM was 9 ± 5 months (IQR, 6–11). Relevant serum biochemical, imaging, and other tests were performed at each elastography test. This study design was approved by the Ethics Committee of the Xijing Hospital of the Air Force Military Medical University.

#### Diagnostic criteria

Referring to the diagnostic criteria established in the 2017 Guidelines [14] for the diagnosis and treatment of PBC, PBC can be diagnosed by meeting any two of the following three criteria:

1. Elevated ALP, an indicator of cholestasis, and imaging that rules out extrahepatic or intrahepatic bile duct obstruction;
2. Positive anti-mitochondrial antibody (AMA)/AMA-M2 or positive for anti-sp100 and/or anti-gp210;
3. Pathology with typical evidence of PBC.

#### Inclusion criteria

1. Perform at least one elastography test;
2. Meet the diagnostic criteria and be at least 18 years of age;
3. Standardized access to UDCA treatment.

#### Exclusion criteria

1. Medication irregularities or the absence of pertinent clinical information;
2. Those with failed or unreliable LSM;
3. Those with combined autoimmune hepatitis, viral hepatitis, primary sclerosing cholangitis, alcoholic liver disease, hepatomegaly, non-alcoholic fatty liver disease, IgG4-associated cholangitis, hemochromatosis, and drug liver injury;
4. Those with a previous decompensated event, a history of malignancy, or another end-stage disease.

### Study methods

#### Clinical data collection

Basic data such as gender, age, and body mass index (BMI) were collected; laboratory indicators such as ALP, GGT, TB, alanine aminotransferase (ALT), AST, albumin (ALB), platelets (PLT), immunoglobulin M (IgM), immunoglobulin G (IgG), and AMA were collected; imaging data such as abdominal ultrasound, electronic gastroduodenoscopy, and electronic computed tomography were collected; and pathological data were also collected.

#### Instruments and measurement methods

Elastography test: The LSM of a candidate with PBC was measured by trained physicians using FibroTouch [15–20] and expressed in kilopascals (kPa). The median of 10 or more consecutive successfully tests was taken as the LSM value. Results with deviation values greater than 1/3 of the median data were considered invalid [21, 22].

Liver pathology assessment: Liver tissue specimens were obtained by ultrasound-guided percutaneous liver puncture and specimens less than 8 mm in total length were considered unacceptable. Pathological assessment was performed independently by two experienced pathologists (at least one of whom was of associate chief medical officer rank or above). Cases, in which there was a disparity in fibrosis grade between the two pathologists, were resolved by consensus agreement. The METAVIR scoring system [23] was used to assess the staging of liver fibrosis in PBC. Stages 0–4 represent normal liver, confluent zone fibrosis, confluent zone fibrosis with minimal bridging fibrosis, bridging fibrosis, and cirrhosis, respectively.

#### Defining the primary outcome

The primary outcome was defined as the occurrence of any of the following events: liver-related death, liver transplantation, or cirrhotic decompensation. Patients with cirrhosis and ascites, hepatic encephalopathy, or variceal bleeding were defined as having cirrhotic decompensation. For patients who have died or received a liver transplant, data were censored at the time of death or transplant. For surviving patients without a liver transplant, data were censored at the onset of cirrhosis-associated complications or at the last follow-up visit. If more than one cirrhosis-related complication occurred during the follow-up period for patients who survived but did not have a liver transplant, data were censored at the first occurrence of a cirrhosis-associated complication. In our study cohort, no patients developed hepatocellular carcinoma (HCC) during follow-up, so we did not identify HCC as a primary outcome.

#### Statistical analysis

R v4.1.3 (Tsinghua, Beijing, China) and SPSS 26.0 (IBM, NY, USA) were employed to analyze the data. Frequencies and percentages, median and IQR and means ± standard deviations were used to describe the data depending on the type of data, and comparisons between groups were made via the Fisher exact probability test, chi-square test, Student-*t* test, or Mann–Whitney *U* test. A generalized linear regression model was used to calculate the amount of change in LSM per unit of time, defined as ΔLSM/ΔT (kPa/year). A univariate Cox regression analysis was performed to screen PBC patients for risk factors for the primary outcomes. Statistically significant variables from the screening results were included in multivariate Cox regression analysis with forward stepwise selection, and the hazard ratio (HR) was determined. LSM progression rate was defined as (LSM at a particular point in time-baseline LSM)/ baseline LSM. Optimal cutoffs for LSM progression rates were calculated using the R language package “survival”. The Kaplan–Meier (K–M) method was used to calculate survival rates without primary outcomes, and the Log-rank and Cox test was used to compare differences in primary outcomes between groups. A two-sided *p* < 0.05 was considered a statistically significant difference.

## Results

### Case screening process and study design

**Fig. 1:**
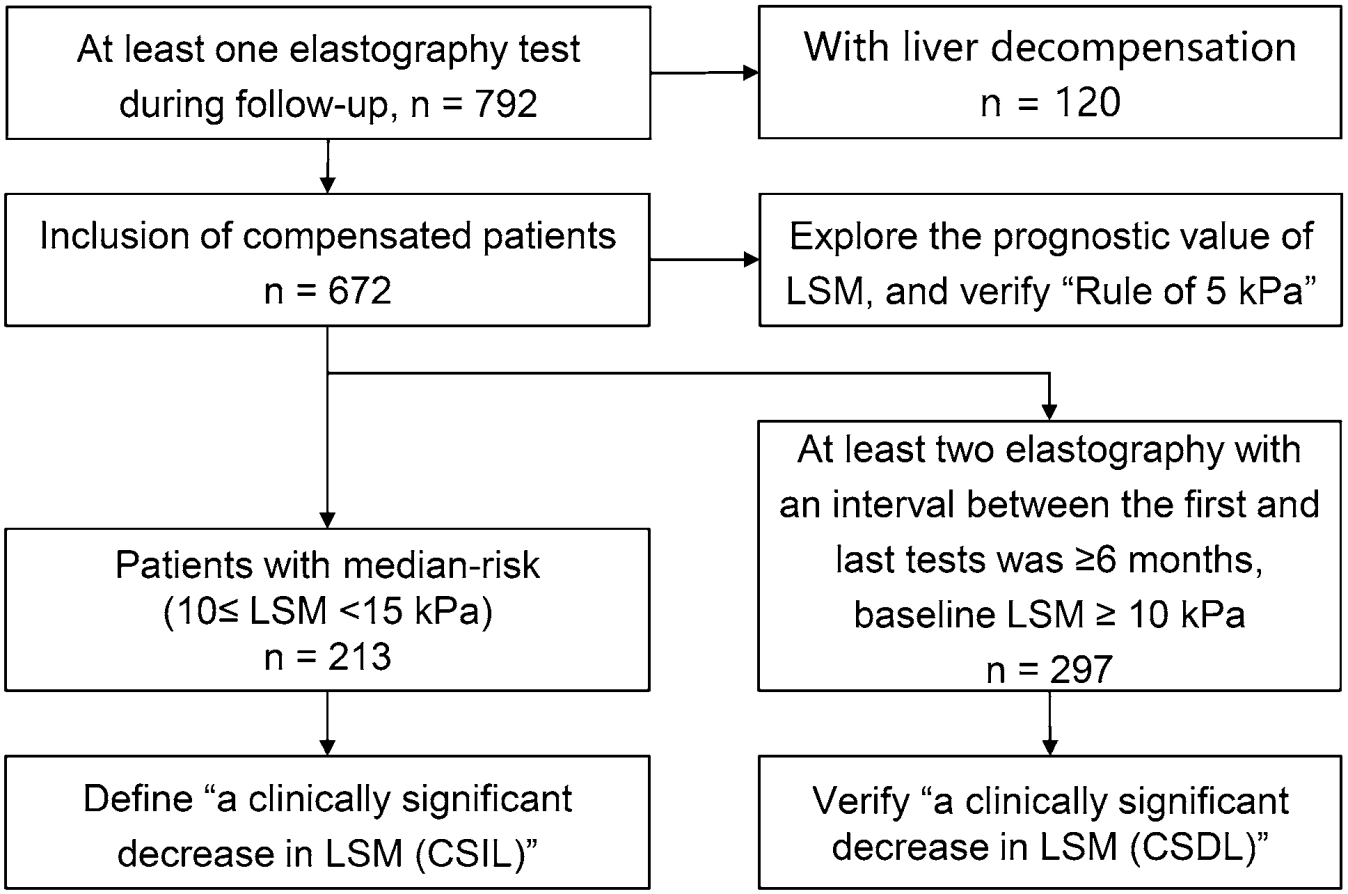
Case screening process and study design. Of these 120 decompensated patients excluded from the study, 75 (63%) had ascites, 43 (36%) had variceal bleeding, and 2 (2%) had hepatic encephalopathy

Figure 1 illustrates the case screening process and study design. The number of patients who underwent elastography testing during the follow-up period was 792, and 120 patients with previous cirrhotic decompensation events were excluded. Finally, 672 patients were included to explore the prognostic value of LSM and validate the “Rule of 5 kPa” and “Relationship between LSM < 10 kPa and prognosis” proposed by Baveno VII. During the follow-up period, a total of 297 patients with compensated advanced chronic liver disease (LSM ≥ 10 kPa) [13] were included to verify “CSDL” proposed by Baveno VII, who met the criteria of “at least two elastography tests during the follow-up period and at least half a year between the first and last test”. 213 patients with median-risk (10 ≤ LSM < 15 kPa) were included to define a clinically significant increase in LSM (CSIL).

### Baseline characteristics of patients with or without the primary outcome

The mean follow-up time for the 672 included patients was 37 ± 14 months. 44 patients (7%) had primary outcomes during follow-up, including 6 (14%) liver-related deaths, 2 (4%) liver transplants, 18 (41%) variceal bleeding, and 18 (41%) ascites. The mean age of the patients at enrollment was 54 ± 9 years; 14% were male; the median BMI was 22.10 kg/m^2^; and 91% were seropositive for AMA or AMA-M2. It was noted that the patients had received UDCA treatment for 28 ± 39 months (IQR, 0–46 months) prior to enrollment, and 244 patients (36%) began UDCA therapy after enrollment.

The baseline data of patients recorded at the first LSM are shown in Table 1, among whom 3 patients (1%) had missing PLT data, 37 (6%) had missing IgG and IgM data, 532 (79%) had abdominal ultrasound, 438 (65%) had electric gastroduodenoscopy, and 288 (43%) had pathology. Patients with the primary outcomes had shorter follow-up time (*p* < 0.001), a higher incidence of pruritus (*p* = 0.005), higher ALP (*p* = 0.006), ALT (*p* = 0.038), AST (*p* < 0.001), and TB (*p* < 0.001), lower ALB (*p* < 0.001), and PLT (*p* < 0.001), a higher incidence of splenomegaly (*p* < 0.001), and esophageal varices (*p* < 0.001), and higher LSM (*p* < 0.001). Although patients with the primary outcomes had a slightly higher rate of advanced fibrosis, there was no statistical difference.

**Table 1:** Baseline parameters in patients with or without primary outcome

Parameters	Total population *n* = 672	PBC without primary outcome *n* = 628	PBC with primary outcome *n* = 44	*P* value
Age (years)	54 ± 9	54 ± 10	57 ± 9	0.055
Male gender (*n*, %)	97 (14)	86 (14)	11 (25)	0.039
Time (months)	37 ± 14	39 ± 13	20 ± 13	< 0.001
BMI (Kg/m^2^)	22.1 (20.64–24)	22.22 (20.7–24.01)	21.37 (19.75–23.41)	0.127
Fatigue (*n*, %)	302 (45)	278 (44)	24 (55)	0.185
Pruritus (*n*, %)	146 (22)	129 (21)	17 (39)	0.005
ALP × ULN	1.00 (0.73–1.58)	0.99 (0.72–1.57)	1.30 (0.97–1.91)	0.006
GGT × ULN	2.27 (0.98–5.21)	2.19 (0.95–5.13)	3.36 (1.46–5.92)	0.104
ALT × ULN	0.78 (0.49–1.43)	0.75 (0.48–1.43)	1.04 (0.69–1.42)	0.038
AST × ULN	1.03 (0.74–1.69)	1.00 (0.74–1.60)	1.69 (1.00–2.13)	< 0.001
TB × ULN	0.71 (0.54–1.03)	0.69 (0.53–0.97)	1.18 (0.84–2.00)	< 0.001
ALB × LLN	1.07 (0.99–1.13)	1.08 (1.00–1.13)	0.97 (0.85–1.06)	< 0.001
PLT × LLN	1.68 (1.18–2.24)	1.72 (1.27–2.26)	0.90 (0.64–1.35)	< 0.001
IgG × ULN	0.86 (0.72–1.00)	0.86 (0.72–0.99)	0.88 (0.79–1.07)	0.148
IgM × ULN	0.95 (0.63–1.55)	0.94 (0.63–1.50)	1.14 (0.67–1.78)	0.315
AMA + (*n*, %)	610 (91)	571 (91)	39 (89)	0.812
Splenomegaly (*n*, %)	177 (33)	151 (31)	26 (70)	< 0.001
Esophageal varices (*n*, %)	86 (20)	63(16)	23 (68)	< 0.001
Fibrosis stage 3–4 (*n*, %)	53 (18)	46 (17)	7 (37)	0.066
LSM (kPa)	10.14 (7.25–13.65)	9.78 (7.07–13.17)	16.62 (12.23–19.11)	< 0.001
ΔLSM/ΔT (kPa/year)	0.10 (– 0.68 to 0.68)	0.09(– 0.60 to 0.64)	0.23 (–0.91 to 2.31)	0.244
Primary outcome (*n*, %)	44 (7)		44 (100)	
Liver transplantation	2 (4)		2 (4)	
Liver-related death	6 (14)		6 (14)	
Liver decompensation	36 (82)		36 (82)	
Variceal bleeding	18 (41)		18 (41)	
Ascites	18 (41)		18 (41)	

PLT: Available in 669 patients

IgM and IgG: Available in 635 patients

Abdominal ultrasound: Available in 532 patients

Endoscopy: Available in 438 patients

Liver biopsy: Available in 288 patients

*P* values were between PBC with primary outcomes and PBC without primary outcomes

*AMA* anti-mitochondrial antibody, *ALP* alkaline phosphatase, *GGT* gamma-glutamyl transferase, *ALT* alanine aminotransferase, *TB* total bilirubin, *ALB* Albumin, *AST* aspartate aminotransferase, *IgG* immunoglobulin G, *IgM* immunoglobulin M, *PLT* platelets, *ULN* upper limit of normal, *LLN* lower limit of normal, *LSM* liver stiffness measurements

Univariate COX regression analysis showed that age, AST, TB, ALB, PLT, LSM, and ΔLSM/ΔT had a statistically significant effect on the occurrence of the primary outcomes (*p* < 0.05), and further inclusion of these variables in the multivariate Cox regression analysis revealed that PLT (*p* < 0.001, HR: 0.252, 95% confidence interval CI 0.118–0.537) was a protective factor for the occurrence of the primary outcomes, and TB (*p* = 0.018, HR: 1.320, 95% CI 1.050–1.66), LSM (*p* < 0.001, HR: 1.190, 95% CI 1.108–1.278) and ΔLSM/ΔT (*p* < 0.001, HR: 1.582, 95% CI 1.354–1.848) were risk factors for the occurrence of the primary outcomes (Supplementary Table 1).

### Validation of the “Rule of 5 kPa” and “Relationship between LSM < 10 kPa and prognosis” proposed by Baveno VII

**Fig. 2:**
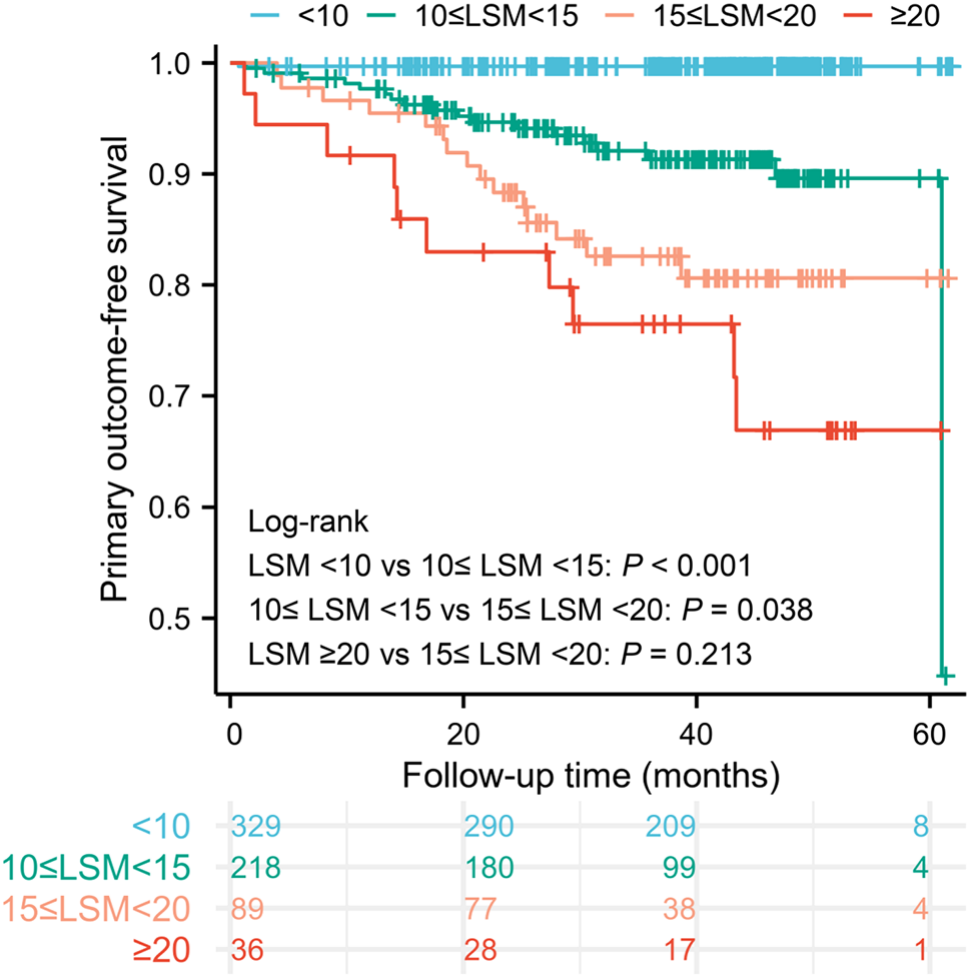
Kaplan–Meier plots of the primary outcome-free survival of patients with PBC based on the rule of 5 kPa (10–15–20 kPa)

The LSM cut-off values of 10, 15, and 20 kPa were used to divide the patients into four groups and the K–M method was used to compare the primary outcome-free survival between the groups (Fig. 2). Log-rank test analysis showed that primary outcome-free survival was statistically significant among the LSM < 10 kPa vs. 10 ≤ LSM < 15 kPa vs. 15 ≤ LSM < 20 kPa groups (*p* < 0.05, all). Limited to sample size, primary outcome-free survival was not statistically significant between 15 ≤ LSM < 20 kPa and LSM ≥ 20 kPa groups (*p = *0.213).

The 3-year risk of the primary outcomes for included patients was 39/468 (8%), with LSM < 10 kPa at 1/240 (< 1%), 10 ≤ LSM < 15 kPa at 16/138 (12%), 15 ≤ LSM < 20 kPa at 14/62 (23%), and LSM ≥ 20 kPa at 8/28 (29%). The LSM 5 kPa scale (10–15–20 kPa) represents a progressively higher risk of liver disease-related death, liver transplantation, or cirrhotic decompensation events. Patients with PBC and LSM < 10 kPa have a negligible 3-year risk of primary outcomes (< 1%).

Considering the negligible 3-year risk of primary outcomes in patients with LSM < 10 kPa and the cut-off value nature of 10 kPa for compensated advanced chronic liver disease [13], and 15 kPa has been well established as the cut-off value for the high-risk group [12]. Therefore, we took baseline LSM threshold values of 10 and 15 kPa to categorize PBC patients into low-, medium-, and high-risk groups (Supplementary Fig. 1).

### Baseline characteristics and prognostic significance of patients with a clinically significant decrease in LSM

Of the 297 patients with compensated advanced chronic liver disease (LSM ≥ 10 kPa) included, 65 progressed from LSM < 10 kPa to ≥ 10 kPa with a mean time of 1.2 ± 0.7 years (IQR, 0.6–1.6 years). At 12-month elastography test, 109 (48%) obtained CSDL (CSDL group) and 120 (52%) did not obtain CSDL (Non-CSDL group). The mean follow-up time for patients enrolled was 40 ± 11 months. A total of 17 (7%) patients had primary outcomes during follow-up, including 3 (18%) liver-related deaths, 1 (6%) liver transplant, 5 (29%) variceal bleeding, and 8 (47%) ascites. The mean age of the patients at enrollment was 55 ± 10 years, 14% were male, the median BMI was 22.04 kg/m^2^, and 92% were seropositive for AMA or AMA-M2.

Assessed at 12-month elastography test, patients were divided into CSDL and Non-CSDL groups based on whether they had acquired CSDL or not. Clinical data for included patients are shown in Table 2, of which 1 (< 1%) had missing PLT data, 14 (6%) had missing IgG and IgM data, 189 (83%) had abdominal ultrasound, 158 (69%) had electric gastroduodenoscopy, and 120 (52%) had pathology. The Non-CSDL group had shorter follow-up time (*p* < 0.001), higher ALP (*p* = 0.011), GGT (*p* = 0.012), and TB (*p* = 0.001), lower PLT (*p* = 0.001), higher incidence of splenomegaly and esophageal varices (*p* = 0.001, both), and higher ΔLSM/ΔT (*p* < 0.001). There was a significant difference in the incidence of primary outcomes between the two groups (*p* < 0.001).

**Table 2:** Parameters in patients with and without a clinically significant decrease in LSM

Parameters	Total population *n* = 229	CSDL *n* = 109	Non-CSDL *n* = 120	*P* value
Age (years)	55 ± 10	54 ± 9	55 ± 10	0.379
Male gender (*n*, %)	31 ( 14)	14 (13)	17 (14)	0.770
Time (months)	40 ± 11	43 ± 10	38 ± 12	< 0.001
BMI (Kg/m^2^)	22.04 (20.03–23.63)	22.39 (20.69–23.88)	21.56 (19.92–23.44)	0.105
Fatigue (*n*, %)	119 (52)	56 (51)	63 (53)	0.865
Pruritus (*n*, %)	60 (26)	24 (22)	36 (30)	0.170
ALP × ULN	1.09 (0.75–1.70)	1.01 (0.67–1.46)	1.26 (0.82–1.90)	0.011
GGT × ULN	2.69 (1.24–5.44)	2.04 (1.11–4.76)	3.26 (1.59–6.59)	0.012
ALT × ULN	0.90 (0.53–1.65)	0.85 (0.50–1.53)	0.95 (0.56–1.68)	0.529
AST × ULN	1.17 (0.83–1.97)	1.06 (0.80–1.89)	1.30 (0.86–2.00)	0.140
TB × ULN	0.74 (0.54–1.06)	0.64 (0.51–0.88)	0.84 (0.58–1.18)	0.001
ALB × LLN	1.05 (0.98–1.12)	1.06 (0.98–1.13)	1.05 (0.97–1.10)	0.327
PLT × LLN	1.56 (1.12–2.22)	1.84 (1.27–2.36)	1.38 (0.99–1.97)	0.001
IgG × ULN	0.87 (0.73–1.03)	0.88 (0.72–1.06)	0.86 (0.74–1.03)	0.760
IgM × ULN	1.03 (0.68–1.63)	1.04 (0.70–1.66)	1.02 (0.63–1.56)	0.820
AMA + (*n*, %)	210 (92)	100 (92)	110 (92)	0.983
Splenomegaly (*n*, %)	75/189 (40)	23/85 (27)	52/104 (50)	0.001
Esophageal varices (*n*, %)	39/158 (25)	10/77 (13)	29/81 (36)	0.001
Fibrosis stage 3–4 (*n*, %)	27/120 (23)	10/56 (18)	17/64 (27)	0.255
LSM (kPa)	12.65 (11.28–15.76)	12.67 (11.03–15.76)	12.65 (11.63–14.86)	0.657
ΔLSM/ΔT (kPa/year)	−0.25 (−1.32–0.70)	−1.04 (−2.29–0.05)	0.33 (−0.46–1.11)	< 0.001
Primary outcome (*n*, %)	17 (7)	0 (0)	17 (14)	< 0.001
Liver transplantation	1 (6)	0 (0)	1 (6)	
Liver-related death	3 (18)	0 (0)	3 (18)	
Liver decompensation	13 (76)	0 (0)	22 (76)	
Variceal bleeding	5 (38)	0 (0)	8 (32)	
Ascites	8 (62)	0 (0)	14 (68)	

PLT: Available in 228 patients

IgM and IgG: Available in 215 patients

Abdominal ultrasound: available in 189 patients

Endoscopy: available in 158 patients

Liver biopsy: available in 120 patients

*P* values were between patients with CSDL and Non-CSDL

*AMA* anti-mitochondrial antibody, *ALP* alkaline phosphatase, *GGT* gamma-glutamyl transferase, *ALT* alanine aminotransferase, *TB* total bilirubin, *ALB* Albumin, *AST* aspartate aminotransferase, *IgG* immunoglobulin G, *IgM* immunoglobulin M, *PLT* platelets, *ULN* upper limit of normal, *LLN* lower limit of normal, *CSDL* a clinically significant decrease in LSM, *LSM* liver stiffness measurements

**Fig. 3:**
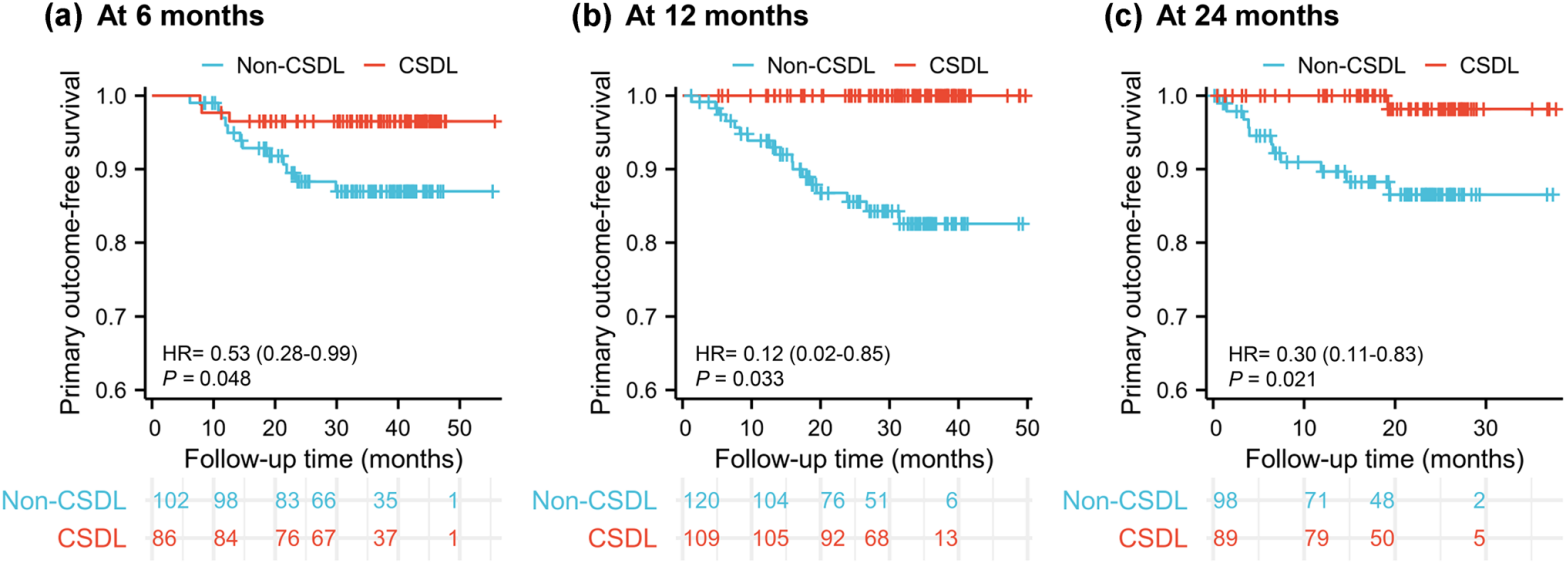
Kaplan–Meier plots of the primary outcome-free survival of patients with a clinically significant decrease in LSM (CSDL) and without CSDL. **a** Evaluated at 6-month elastography test; **b** Evaluated at 12 months; **c** Evaluated at 24 months

Evaluated at 6 months, 86 (46%) obtained CSDL and 102 (54%) did not obtain CSDL. The average evaluation time was 8 ± 5 months (IQR, 6–8) and 7 ± 4 months (IQR, 5–8), respectively (*p* = 0.575). The number of primary outcomes was 3 (3%) and 12 (12%), respectively. Cox analysis showed that the primary outcome-free survival was statistically significant (*p* = 0.048, Fig. 3a). CSDL reduced the risk of the primary outcomes in patients with PBC by 0.53-fold (95% CI 0.281–0.994).

Evaluated at 12 months, 109 (48%) obtained CSDL and 120 (52%) did not obtain CSDL. The average evaluation time was 14 ± 4 months (IQR, 11–16) and 16 ± 6 months (IQR, 11–16), respectively (*p* = 0.085). The number of primary outcomes was 0 (0%) and 17 (14%), respectively. Cox analysis showed that the primary outcome-free survival was statistically significant (*p* < 0.001, Fig. 3b). CSDL reduced the risk of the primary outcomes in patients with PBC by 0.12-fold (95% CI 0.017–0.847).

Evaluated at 24 months, 89 (48%) obtained CSDL and 98 (52%) did not obtain CSDL. The average evaluation time was 24 ± 5 months (IQR, 20–26) and 25 ± 7 months (IQR, 22–28), respectively (*p* = 0.135). The number of primary outcomes was 1 (1%) and 11 (11%), respectively. Cox analysis showed that the primary outcome-free survival was statistically significant (*p* = 0.021, Fig. 3c). CSDL reduced the risk of the primary outcomes in patients with PBC by 0.30-fold (95% CI 0.108–0.834).

### Defining a clinically significant increase in LSM in median-risk patients

Median-risk (10 ≤ LSM < 15 kPa) patients were equivalent to a T-junction and were more likely to progress to the high-risk group as compared to low-risk patients or to regress to the low-risk group during treatment as compared to high-risk patients. We previously verified CSDL to assess remission, and now we try to define CSIL to evaluate progression. Since LSM ≥ 15 kPa can be used to define high-risk patients, we tried to define LSM progression to 15 kPa as the progression group. Of the 213 patients included with 10 ≤ LSM < 15 kPa, 65 progressed from LSM < 10 kPa to ≥ 10 kPa with a mean time of 14 ± 8 months (IQR, 7–19), and no patient with LSM < 10 progressed directly to ≥ 15 without at least one LSM monitoring with 10 ≤ LSM < 15 kPa. Evaluated at 6, 12, and 24 months, Cox analysis showed that the primary outcome-free survival was all statistically significant (*p* < 0.05, all; Supplementary Fig. 2).

Further, we try to define the optimal LSM progression rate (ΔLSM/baseline LSM). At 12-month elastography test, we calculated the LSM progression rate. Univariate and multivariate Cox regression analysis revealed that LSM progression rate was an independent risk factor for the occurrence of the primary outcomes (*p* < 0.05; Supplementary Table 2), and the optimal cut-off value was 21.58% (Supplementary Fig. 3). Therefore, we took 20% as the optimal LSM progression rate. Evaluated at 6, 12, and 24 months, Cox analysis showed that the primary outcome-free survival was all statistically significant (*p* < 0.05, all; Supplementary Fig. 4).

**Fig. 4:**
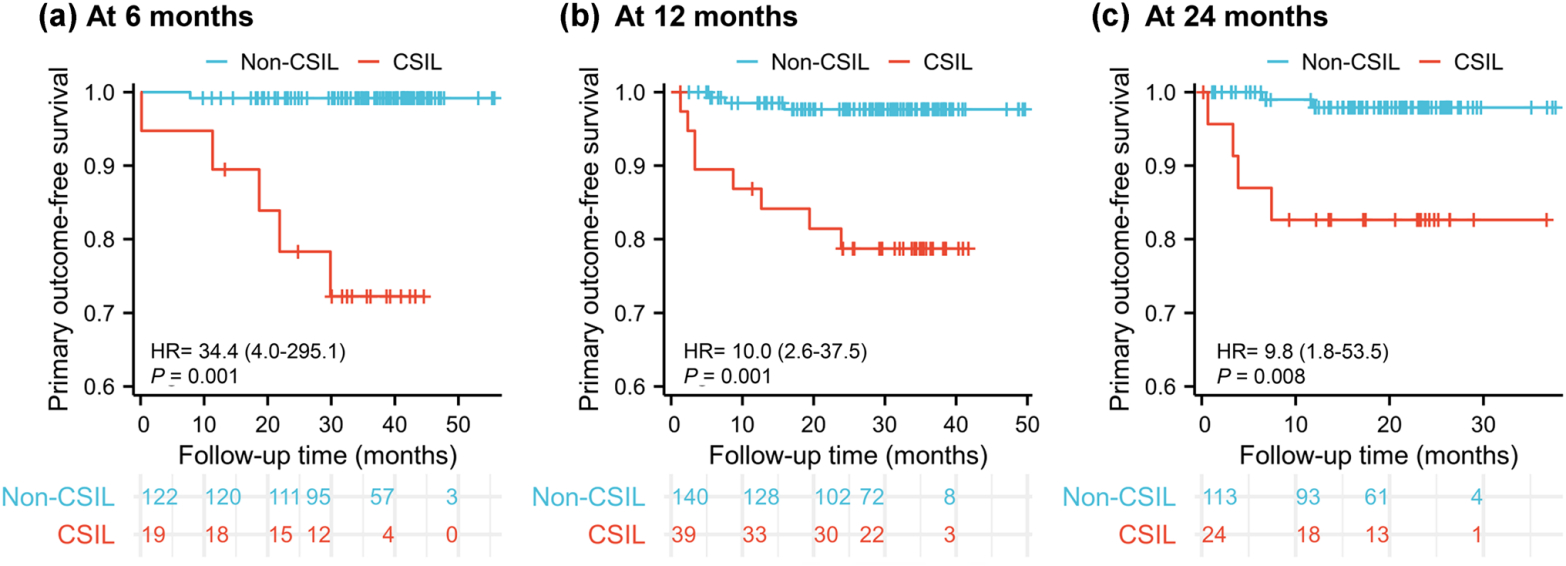
Kaplan–Meier plots of the primary outcome-free survival of median-risk (10 ≤ LSM < 15 kPa) patients with a clinically significant increase in LSM (CSIL) and without CSIL. **a** Evaluated at 6-month elastography test; **b** Evaluated at 12 months; **c** Evaluated at 24 months

Then we defined CSIL as a progression of at least 20% or to more than 15 kPa in LSM. Evaluated at 6 months, 19 (13%) obtained CSIL and 122 (87%) did not obtain CSIL. The average evaluation time was 6 ± 2 months (IQR, 6–7) and 7 ± 1 months (IQR, 6–7), respectively (*p* = 0.904). The number of primary outcomes was 5 (26%) and 1 (1%), respectively. Cox analysis showed that the primary outcome-free survival was statistically significant (*p* = 0.001, Fig. 4a). CSIL increased the risk of the primary outcomes in patients with PBC by 34.47-fold (95% CI 4.026–295.117).

Evaluated at 12 months, 39 (22%) obtained CSIL and 140 (78%) did not obtain CSIL. The average evaluation time was 13 ± 2 months (IQR, 11–16) and 13 ± 2 months (IQR, 12–16), respectively (*p* = 0.855). The number of primary outcomes was 8 (21%) and 3 (2%), respectively. Cox analysis showed that the primary outcome-free survival was statistically significant (*p* = 0.001, Fig. 4b). CSIL increased the risk of the primary outcomes in patients with PBC by 9.96-fold (95% CI 2.640–37.545).

Evaluated at 24 months, 24 (18%) obtained CSIL and 113 (82%) did not obtain CSIL. The average evaluation time was 24 ± 7 months (IQR, 20–26) and 25 ± 5 months (IQR, 20–29), respectively (*p* = 0.101). The number of primary outcomes was 4 (17%) and 2 (2%), respectively. Cox analysis showed that the primary outcome-free survival was statistically significant (*p* = 0.008, Fig. 4c). CSIL increased the risk of the primary outcomes in patients with PBC by 9.79-fold (95% CI 1.793–53.474).

## Discussion

In this study, we assessed the prognostic value of LSM in patients with PBC and validated the “novel concept” proposed by Baveno VII. It was found that: (1) LSM and ΔLSM/ΔT were independent risk factors for primary outcomes; (2) the LSM 5 kPa scale (10–15–20 kPa) represented a progressively higher risk of the primary outcomes; (3) patients with LSM < 10 kPa have a low risk of primary outcomes at 3 years (< 1%); (4) cut-off values of 10 and 15 kPa can be used to classify patients into low-, medium-, and high-risk groups; (5) a clinically significant decrease in LSM was associated with a significantly lower risk of primary outcomes; and (6) a clinically significant increase in LSM was associated with substantially raised risk of primary outcomes.

Second-line drug therapy (obeticholic acid or fibrates) is recommended for PBC patients with a poor UDCA response, regardless of disease stage [14, 24]. The finding that elevated LSM and ΔLSM/ΔT were independent risk factors for poor prognosis in PBC was largely consistent with the finding of Corpechot et al. [11], provides clues for early identification of patients with poor response and timely initiation of second-line drug therapy, although more clinical data are needed to determine the optimal prognostic cut-off. At the same time, this study found that LSM and ΔLSM/ΔT were more effective in assessing the prognosis of PBC patients than the classical indicators of ALP and TB. Future attempts should be made to integrate LSM and/or ΔLSM/ΔT into biochemical response criteria, which may optimize the effectiveness of prognosis assessment in PBC patients.

The heterogeneity of disease progression in patients with PBC poses a serious challenge for fine-tuned management. An international multicenter study [11] of 3985 PBC patients from 12 countries found that baseline LSM cut-off values of 8 and 15 kPa were used to classify PBC patients into low-, medium-, and high-risk groups. The present study confirmed the “Rule of 5 kPa” (10–15–20 kPa) based on LSM, which represents a progressively higher relative risk of the primary outcomes, and facilitates the early implementation of individualized interventions for patients in different risk strata.

Our study confirmed that CSDL, defined as a reduction in liver stiffness of at least 20% and < 20 kPa, or to less than 10 kPa, can be evaluated at 6-, 12-, and 24-month elastography tests, and was associated with a significant reduction in the risk of the primary outcomes, suggesting that it could be used as a substitute for the primary endpoint event in clinical trials. This is supported by the results of the recent phase III bezafibrate trial in patients with PBC who responded poorly to UDCA (BEZURSO). Patients in the placebo group had a significant increase in LSM, while patients in the bezafibrate group had a stable or even reduced LSM [25].

We further defined CSIL to evaluate the progression in patients with median risk or low. Our study demonstrated that CSIL, defined as an increase in LSM of ≥  + 20% or any increase to LSM ≥ 15 kPa, was associated with a substantially raised risk of primary outcomes, which can be evaluated at 6-, 12-, and 24-month elastography tests.

According to these findings, treatment and monitoring strategies based on LSM were established. Baseline LSM values of 10 and 15 kPa divide patients into low-, median-, and high-risk groups. For patients with median to high risk, CSDL can dynamically assess the disease remission status under current treatment; for patients with median risk or who progress from low to median risk, CSIL can dynamically evaluate the disease progression. Their clinical application may optimize the clinical monitoring and medication strategy for PBC patients.

The study was a single-center retrospective cohort study with some bias in the selection of patients; the follow-up cohort included a high proportion of early-stage (F0–F2) patients with a short follow-up period and a low proportion of patients with primary outcomes; given the small number of patients with paired liver biopsies and varying time intervals, it is difficult to evaluate the relationship between progression or remission of liver stiffness and pathological results; the patients included were a mixed group of primary and treated patients and could have affected the results. Future validation should be conducted in a multicenter, prospective, large sample size, and long-term follow-up clinical cohort study.

In summary, LSM can be used to monitor disease progression and predict long-term prognosis in patients with PBC.

### Supplementary Information

Below is the link to the electronic supplementary material.

Supplementary file1 (DOCX 2954 KB)

## Data Availability

The data that support the findings of this study are available on request from the corresponding author.

## Abbreviations

PBC: Primary biliary cholangitis
UDCA: Ursodeoxycholic acid
HR: Hazard ratio
CI: Confidence interval
AMA: Anti-mitochondrial antibody
ALP: Alkaline phosphatase
GGT: Gamma-glutamyl transpeptidase
ALT: Alanine transaminase
AST: Aspartate transaminase
TB: Total bilirubin
ALB: Albumin
PLT: Platelet
IgG: Immunoglobulin G
IgM: Immunoglobulin M
K–M: Kaplan–Meier
ULN: Upper limit of the normal range
LLN: Lower limit of the normal range
LSM: Liver stiffness measurement
CSDL: A clinically significant decrease in LSM
CSIL: A clinically significant increase in LSM
UK: United Kingdom
IQR: Interquartile ranges
BMI: Body mass index
F: Fibrosis stage

## References

[1] Shang Y, Leung PSC, Gershwin ME (2022). Primary biliary cholangitis: personalized medicine for optimal therapeutic opportunities. Sci Bull (Beijing).

[2] Azemoto N, Abe M, Murata Y (2009). Early biochemical response to ursodeoxycholic acid predicts symptom development in patients with asymptomatic primary biliary cirrhosis. J Gastroenterol.

[3] Parés A, Caballería L, Rodés J (2006). Excellent long-term survival in patients with primary biliary cirrhosis and biochemical response to ursodeoxycholic acid. Gastroenterology.

[4] Corpechot C, Abenavoli L, Rabahi N (2008). Biochemical response to ursodeoxycholic acid and long-term prognosis in primary biliary cirrhosis. Hepatology.

[5] Corpechot C, Chazouilleres O, Poupon R (2011). Early primary biliary cirrhosis: biochemical response to treatment and prediction of long-term outcome. J Hepatol.

[6] Kuiper EMM, Hansen BE, de Vries RA (2009). Improved prognosis of patients with primary biliary cirrhosis that have a biochemical response to ursodeoxycholic acid. Gastroenterology.

[7] Carbone M, Sharp SJ, Flack S (2016). The UK-PBC risk scores: derivation and validation of a scoring system for long-term prediction of end-stage liver disease in primary biliary cholangitis. Hepatology.

[8] Lammers WJ, Hirschfield GM, Corpechot C (2015). Development and validation of a scoring system to predict outcomes of patients with primary biliary cirrhosis receiving ursodeoxycholic acid therapy. Gastroenterology.

[9] Kumagi T, Guindi M, Fischer SE (2010). Baseline ductopenia and treatment response predict long-term histological progression in primary biliary cirrhosis. Am J Gastroenterol.

[10] Yang C, Guo G, Li B (2023). Prediction and evaluation of high-risk patients with primary biliary cholangitis receiving ursodeoxycholic acid therapy: an early criterion. Hepatol Int.

[11] Corpechot C, Carrat F, Poujol-Robert A (2012). Noninvasive elastography-based assessment of liver fibrosis progression and prognosis in primary biliary cirrhosis. Hepatology.

[12] Corpechot C, Carrat F, Gaouar F (2022). Liver stiffness measurement by vibration-controlled transient elastography improves outcome prediction in primary biliary cholangitis. J Hepatol.

[13] de Franchis R, Bosch J, Garcia-Tsao G (2022). Baveno VII – Renewing consensus in portal hypertension. J Hepatol.

[14] Hirschfield GM, Beuers U, Corpechot C (2017). EASL clinical practice guidelines: the diagnosis and management of patients with primary biliary cholangitis. J Hepatol.

[15] Man S, Deng Y, Ma Y (2023). Prevalence of liver steatosis and fibrosis in the general population and various high-risk populations: a nationwide study with 5.7 million adults in China. Gastroenterology.

[16] Liu Y, Guo G, Zheng L (2023). Effectiveness of fenofibrate in treatment-naive patients with primary biliary cholangitis: a randomized clinical trial. Am J Gastroenterol.

[17] Xu Y, Liu Y, Cao Z (2019). Comparison of FibroTouch and FibroScan for staging fibrosis in chronic liver disease: single-center prospective study. Dig Liver Dis.

[18] Zuo Z, Cui H, Wang M (2022). Diagnostic of FibroTouch and six serological models in assessing the degree of liver fibrosis among patients with chronic hepatic disease: a single-center retrospective study. PLoS One.

[19] Qu Y, Song Y, Chen C (2021). Diagnostic performance of fibrotouch ultrasound attenuation parameter and liver stiffness measurement in assessing hepatic steatosis and fibrosis in patients with nonalcoholic fatty liver disease. Clin Transl Gastroenterol.

[20] Peng X, Tian A, Li J (2022). Diagnostic value of fibrotouch and non-invasive fibrosis indexes in hepatic fibrosis with different aetiologies. Dig Dis Sci.

[21] Ferraioli G, Maiocchi L, Lissandrin R (2020). Accuracy of the elastPQ^®^ technique for the assessment of liver fibrosis in patients with chronic hepatitis c: a “real life” single center study. J Gastrointestin Liver Dis.

[22] Fang C, Jaffer OS, Yusuf GT (2018). Reducing the number of measurements in liver point shear-wave elastography: factors that influence the number and reliability of measurements in assessment of liver fibrosis in clinical practice. Radiology.

[23] Bedossa P, Poynard T (1996). An algorithm for the grading of activity in chronic hepatitis C. The METAVIR Cooperative Study Group. Hepatology.

[24] Lindor KD, Bowlus CL, Boyer J (2018). Primary biliary cholangitis: 2018 practice guidance from the american association for the study of liver diseases. Hepatology.

[25] Corpechot C, Chazouilleres O, Rousseau A (2018). A placebo-controlled trial of bezafibrate in primary biliary cholangitis. N Engl J Med.

