# Supplementary material for: Prognostic significance of liver stiffness in patients with primary biliary cholangitis: validation of Baveno VII criteria
Source: Hepatol Int. 2023 Sep 19;18(1):206–15. doi: 10.1007/s12072-023-10587-w (PMC10857967; doi:10.1007/s12072-023-10587-w)
Supplement: Supplementary file 1 — Supplementary file1 (DOCX 2954 KB) [file 12072_2023_10587_MOESM1_ESM.docx]

**Supplementary Table**

| Supplementary Table 1. Analysis of risk factors for primary outcomes in the whole cohort. | | | | | |  |
| --- | --- | --- | --- | --- | --- | --- |
| Parameters | Univariate analysis | | | Multivariate analysis | | |
|  | Chi square | HR (95% CI) | *P* value | Chi square | HR (95% CI) | *P* value |
| Male gender | 3.072 | 1.842(0.930-3.648) | 0.080 |  |  |  |
| Age (years) | 4.209 | 1.034(1.001-1.067) | 0.040 |  |  |  |
| ALP×ULN | 3.811 | 1.210(0.999-1.465) | 0.051 |  |  |  |
| AST×ULN | 9.016 | 1.211(1.069-1.373) | 0.003 |  |  |  |
| TB×ULN | 23.928 | 1.402(1.224-1.605) | < 0.001 | 5.569 | 1.320(1.050-1.660) | 0.018 |
| ALB×LLN | 46.139 | 0.001(0.000-0.006) | < 0.001 |  |  |  |
| PLT×LLN | 30.534 | 0.217(0.129-0.364) | < 0.001 | 10.233 | 0.252(0.118-0.537) | < 0.001 |
| LSM (kPa) | 48.916 | 1.109(1.077-1.142) | < 0.001 | 22.652 | 1.190(1.108-1.278) | < 0.001 |
| ΔLSM/ΔT (kPa/year) | 16.507 | 1.497(1.232-1.819) | < 0.001 | 33.594 | 1.582(1.354-1.848) | < 0.001 |
| ΔLSM/ΔT is the amount of change in LSM per unit of time.  *P* values and hazard ratios were calculated by stepwise Cox regression analysis.  Abbreviations: ALP, alkaline phosphatase; TB, total bilirubin; ALB, Albumin; AST, aspartate-aminotransferase; PLT, platelets; ULN, upper limit of normal; LLN, lower limit of normal; LSM, liver stiffness measurements; CI, confidence interval; HR, hazard ratios. | | | | | |  |

| Supplementary Table 2. Analysis of risk factors for primary outcomes in patients with median-risk. | | | | | |  |
| --- | --- | --- | --- | --- | --- | --- |
| Parameters | Univariate analysis | | | Multivariate analysis | | |
|  | Chi square | HR (95% CI) | *P* value | Chi square | HR (95% CI) | *P* value |
| GGT×ULN | 5.646 | 1.074(1.013-1.139) | 0.017 |  |  |  |
| ALB×LLN | 4.208 | 0.001(0.000-0.748) | 0.040 |  |  |  |
| TB×ULN | 3.891 | 1.470(1.002-2.156) | 0.049 | 4.451 | 1.604(1.034-2.488) | 0.035 |
| PLT×LLN | 7.838 | 0.216(0.074-0.631) | 0.005 | 5.179 | 0.190(0.046-0.794) | 0.023 |
| ΔLSM/baseline LSM | 19.183 | 1.030(1.016-1.044) | <0.001 | 13.086 | 1.027(1.012-1.041) | <0.001 |
| *P* values and hazard ratios were calculated by stepwise Cox regression analysis.  Abbreviations: ALP, alkaline phosphatase; TB, total bilirubin; ALB, Albumin; AST, aspartate-aminotransferase; PLT, platelets; ULN, upper limit of normal; LLN, lower limit of normal; LSM, liver stiffness measurements; CI, confidence interval; HR, hazard ratios. | | | | | |  |

| Supplementary Table 3. Baseline and 12-months parameters in patients with or without CSDL. | | | | | | | | | |
| --- | --- | --- | --- | --- | --- | --- | --- | --- | --- |
| Parameters | | PLT×LLN | ALT×ULN | AST× ULN | ALB×LLN | TB× ULN | ALP× ULN | GGT× ULN | IgM× ULN |
| Non-NSDL (n=100) | Baseline | 1.38 | 1.00 | 1.33 | 1.05 | 0.84 | 1.23 | 3.56 | 1.04 |
|  | 12-month | 1.92 | 0.85 | 1.06 | 1.07 | 0.62 | 1.01 | 2.11 | 1.05 |
|  | Difference | 0.54 | -0.15 | -0.27 | 0.02 | -0.22 | -0.22 | -1.45 | 0.01 |
| NSDL (n=92) | Baseline | 1.43 | 0.73 | 1.00 | 1.10 | 0.85 | 1.03 | 2.99 | 0.91 |
|  | 12-month | 1.95 | 0.55 | 0.80 | 1.10 | 0.62 | 0.81 | 1.12 | 0.90 |
|  | Difference | 0.52 | -0.18 | -0.20 | 0.00 | -0.23 | -0.22 | -1.87 | -0.01 |

The table shows the median of each parameter.

Median differences in each parameter were calculated by the 12-month value minus the baseline value.

No significant difference was found in difference of each parameter between NSDL and Non-NSDL groups.

Abbreviations: ALP, alkaline phosphatase; GGT, gamma-glutamyl transferase; ALT, alanine-aminotransferase; TB, total bilirubin; ALB, Albumin; AST, aspartate-aminotransferase; IgM, immunoglobulin M; PLT, platelets; ULN, upper limit of normal; LLN, lower limit of normal; CSDL, a clinically significant decrease in LSM; LSM, liver stiffness measurements

| Supplementary Table 4. Pearson correlation analysis of CSDL and clinical data. | | | | | | | | | |
| --- | --- | --- | --- | --- | --- | --- | --- | --- | --- |
| Parameters | | Difference (n=192) | | | | | | | |
|  |  | PLT | ALT | AST | ALB | TB | ALP | GGT | IgM |
| CSDL | *P* value | 0.831 | 0.130 | 0.047 | 0.155 | 0.213 | 0.662 | 0.307 | 0.205 |
|  | Coefficient | 0.016 | -0.110 | -0.143 | 0.103 | -0.090 | 0.032 | 0.074 | 0.093 |

Difference (PLT, AST, ALT, ALB, TB, ALP, GGT, and IgM) was calculated by the 12-month value minus the baseline value. Correlation analysis was conducted between the differences of each clinical data of each person and whether CSDL was obtained.

Abbreviations: ALP, alkaline phosphatase; GGT, gamma-glutamyl transferase; ALT, alanine-aminotransferase; TB, total bilirubin; ALB, Albumin; AST, aspartate-aminotransferase; IgM, immunoglobulin M; PLT, platelets; ULN, upper limit of normal; LLN, lower limit of normal; CSDL, a clinically significant decrease in LSM; LSM, liver stiffness measurements

**Supplementary Figure**

**
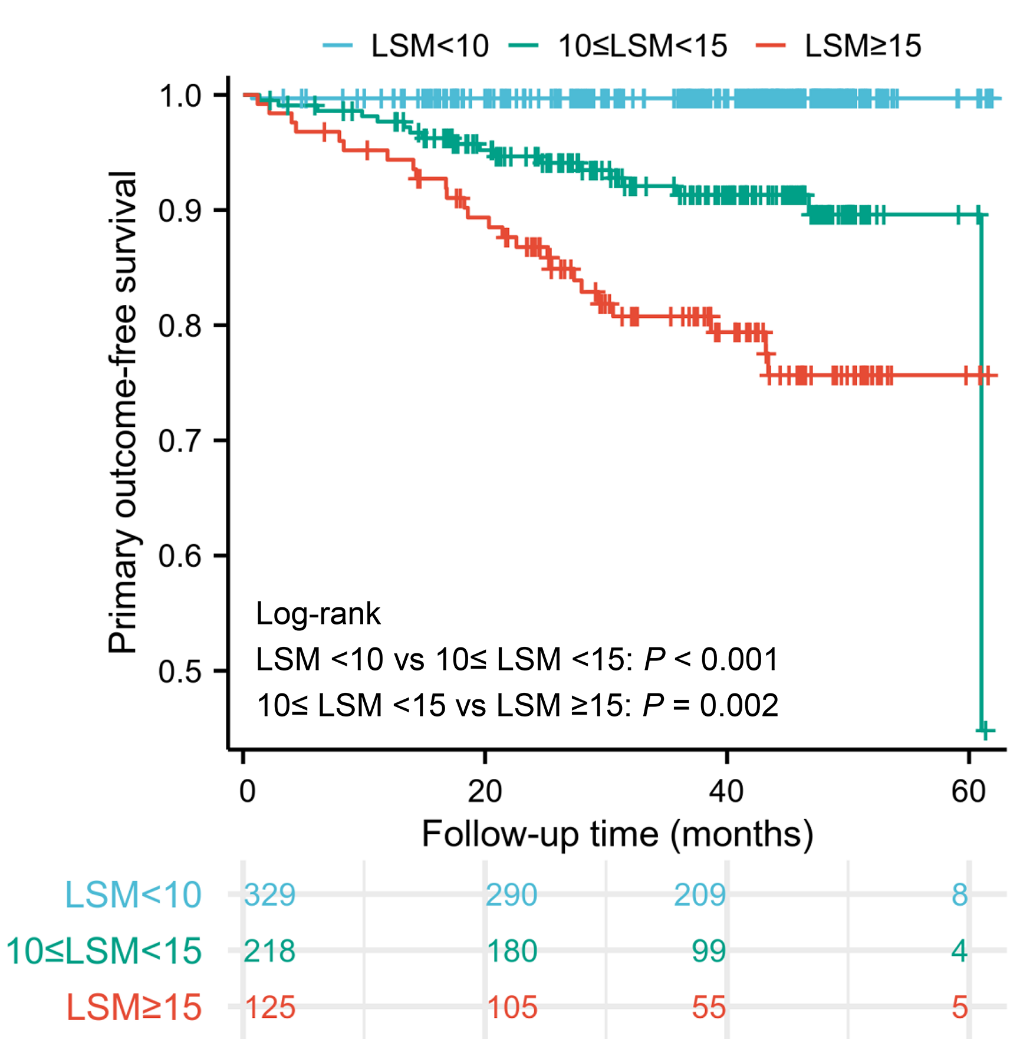
**

**Supplementary Figure 1** Kaplan–Meier plots of the primary outcome-free survival with baseline LSM cut-off values of 10 and 15 kPa.

**
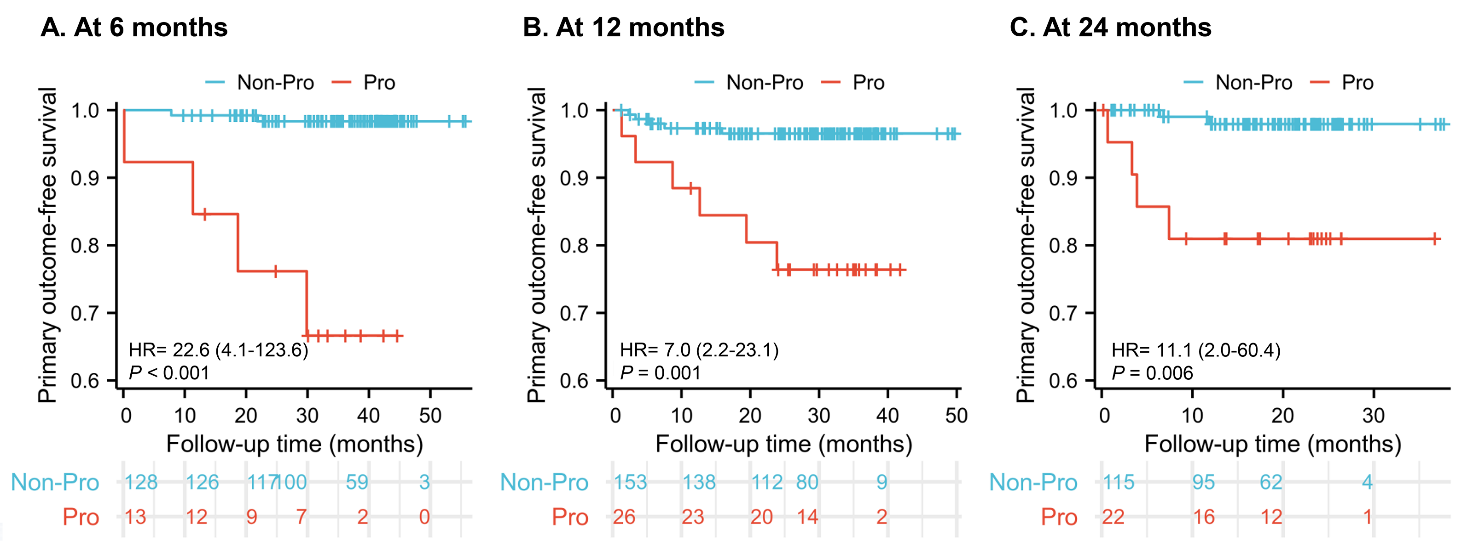
**

**Supplementary Figure 2** Kaplan–Meier plots of the primary outcome-free survival of median-risk patients (10≤ LSM <15 kPa) with (Pro group) or without a progression in LSM to 15 kPa (Non-Pro group). **A** Evaluated at 6-month elastography test; **B** Evaluated at 12-month; **C** Evaluated at 24-month.


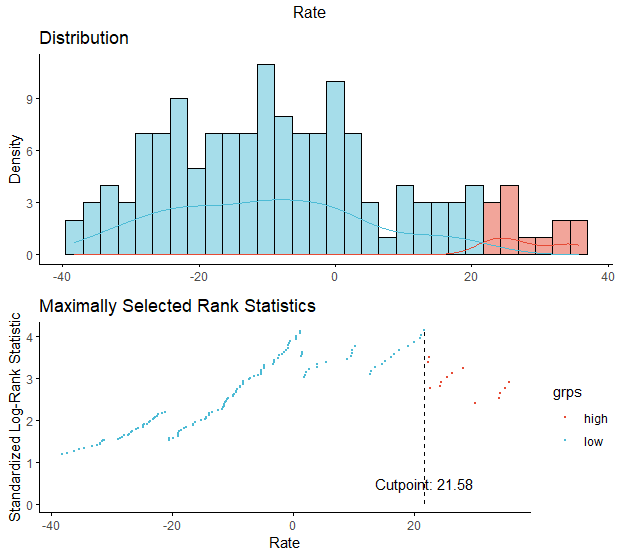
**
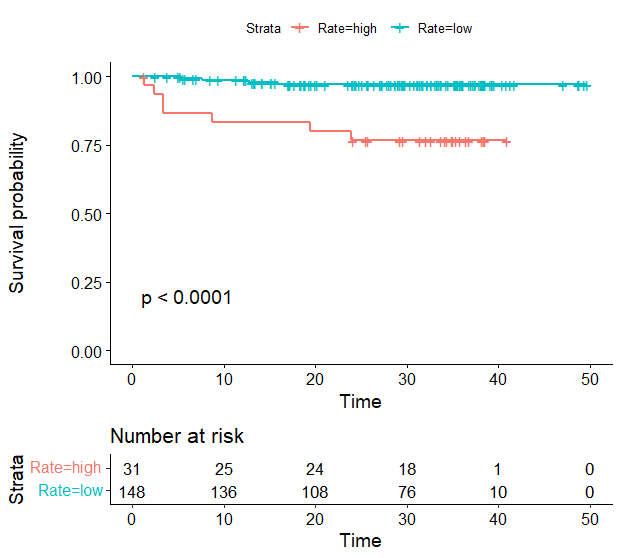
**

**Supplementary Figure 3** Calculated the optimal cutoff value for LSM progression rate using the R language package “survival”.


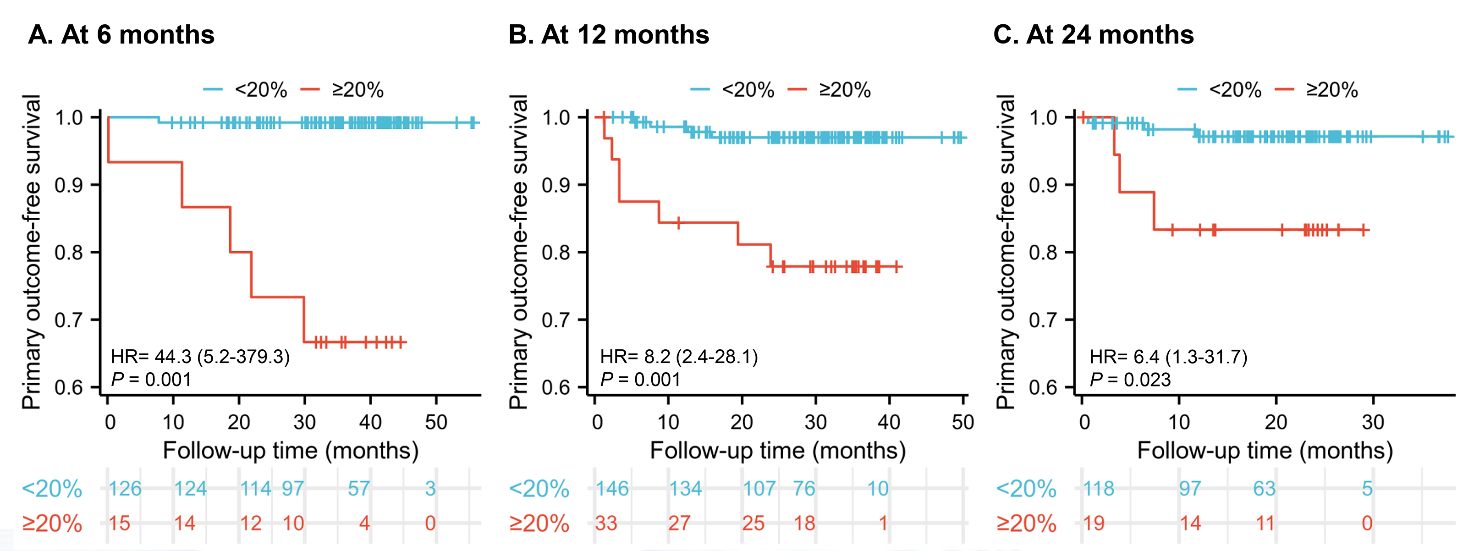


**Supplementary Figure 4** Kaplan–Meier plots of the primary outcome-free survival of median-risk patients (10≤ LSM <15 kPa) with (≥20% group) or without LSM progression rates of ≥ +20% (<20% group). **A** Evaluated at 6-month elastography test; **B** Evaluated at 12-month; **C** Evaluated at 24-month.

**
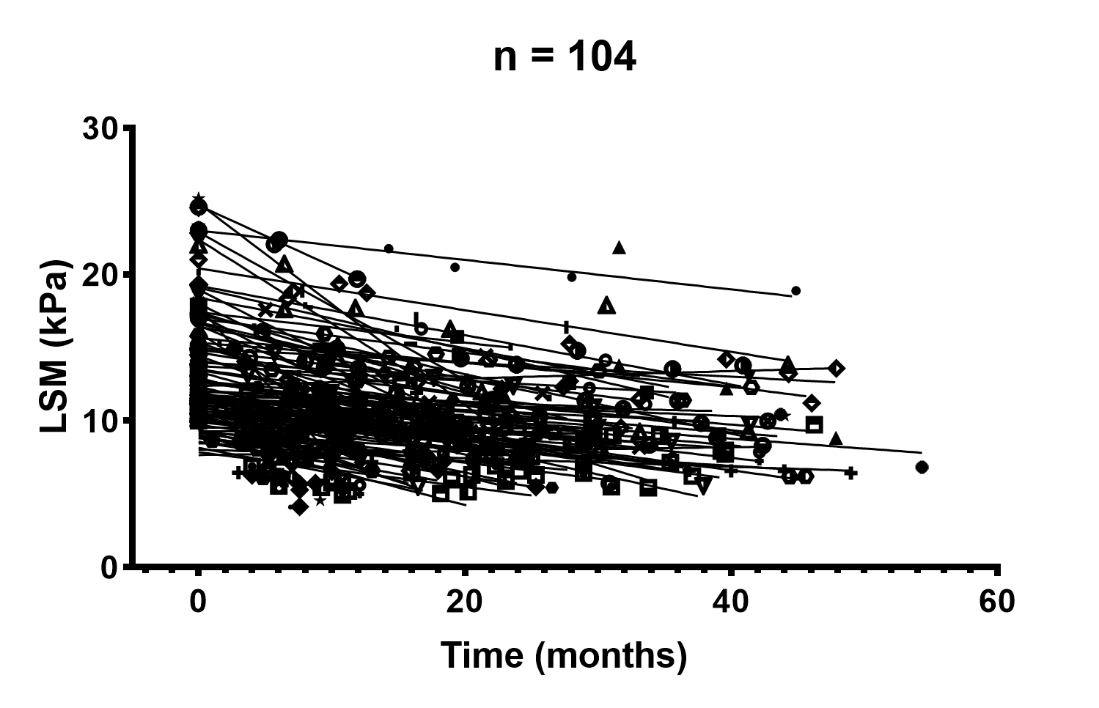
**

**Supplementary Figure 5** Linear regression fitting linear assesses the dynamics of liver stiffness measurements (LSM, at least 3 times) in patients with a clinically significant decrease in LSM. A total of 486 LSM were performed in 104 patients.
